# Comparison of Constitutive Models and Microstructure Evolution of GW103K Magnesium Alloy during Hot Deformation

**DOI:** 10.3390/ma15124116

**Published:** 2022-06-09

**Authors:** Lan Yin, Yunxin Wu

**Affiliations:** 1 Light Alloy Research Institute, Central South University, Changsha 410083, China; yinlan1211@csu.edu.cn; 2 State Key Laboratory of High Performance Complex Manufacturing, Central South University, Changsha 410083, China

**Keywords:** GW103K magnesium alloy, Johnson Cook, strain-compensated Arrhenius, BP neural network, microstructural evolution, hot deformation

## Abstract

The characteristics of constitutive behavior and microstructure evolution of GW103K magnesium alloy were investigated via hot compression tests at a strain rate of 0.001–1 s^−1^ and a temperature of 623–773 K. The rheological stress of GW103K alloy decreased with increasing temperature or decreasing strain rate during hot deformation. Three models including the Johnson Cook (JC) model, the strain-compensated Arrhenius (SCA) model and back-propagation neural networks (BPNN) were applied to describe the constitutive relationships. Subsequently, the predictability and precision of the models were compared by evaluating the correlation coefficient (R), root mean square errors (RMSE), and relative errors (RE). Compared with the JC and SCA models, the BPNN model was more efficient and had higher prediction accuracy in describing flow stress behavior. Furthermore, EBSD maps confirmed that magnesium alloy easily causes dynamic recrystallization (DRX) during hot deformation. The volume fraction and size of DRX grains increased with decreasing strain rate and/or increasing temperature.

## 1. Introduction

As a kind of lightweight structural material, the rare earth magnesium alloy, which has high performance in specific strength, heat conduction, toughness and eco-friendliness, has great potential for use in the automotive, aircraft, 3C electronics and other fields [1,2]. Nevertheless, due to a limited slip system, magnesium alloy is difficult to deform at a low temperature [3,4]. It is necessary to study the hot deformation behavior of magnesium alloy. The deformation parameters (temperature, strain and strain rate) play a critical role in the plastic deformation property of alloys during the hot-forming process [5]. Additionally, deformation processes have a significant influence on the microstructure of alloys, which is not completely understood [6]. Therefore, the constitutive model and microstructure evolution of GW103K magnesium alloy at an elevated temperature are addressed in this paper.

In order to analyze and predict flow behaviors of metals and alloys, many constitutive models have been proposed by researchers. Generally, the constitutive models can be summarized as phenomenological, physical-based and artificial neural network models [7]. Up to now, as representative phenomenological models, the JC equation [8] and Arrhenius [9] equation have been widely employed. The JC constitutive equation with few material constants has been employed on aluminum alloy [10], magnesium alloy [11] and titanium alloy [12]. Similarly, the Arrhenius constitutive model has also been successfully used on a variety of materials [13,14,15]. In addition, the application of the artificial neural network (ANN) method in constitutive relationship prediction has attracted extensive attention. Especially, it is used to solve complex or highly nonlinear problems [16]. In recent years, many researchers have begun to pay attention to the comparative study of several models. For example, Abbasi-Bani [17] compared the capability of the JC and Arrhenius equations in forecasting the flow behaviors of Mg–6Al–1Zn alloy. Adarsh et al. [18] developed the Arrhenius equation, the SCA model, and the ANN model to investigate the elevated temperature behavior of SMAs. Wang et al. [19] employed the Arrhenius model, the JC model and the ANN model to describe the rheological behavior of 20MnNiMo low-carbon alloy.

Additionally, the main softening mechanism of metals and alloys during hot deformation has been confirmed as DRX and dynamic recovery (DRV). DRX has been found to be one of the main deformation mechanisms in materials such as copper, magnesium alloy and stainless steel [20]. DRX has two forms, including discontinuous DRX (DDRX) and continuous DRX (CDRX). Therein, DDRX is the conventional DRX process, in which nucleation occurs at prior grain boundaries and twin boundaries. In this mechanism, nucleation and growth stages can be clearly distinguished [21]. In contrast, the CDRX process involves subgrain rotation near prior grain boundaries when the material is strained. This leads to a misorientation gradient from the center to the edge of the parent grains [22,23]. The local misorientation on the grain boundary further increases under larger strains. Subsequently, low-angle grain boundaries (LAGBs) develop high-angle grain boundaries (HAGBs) [24].

In this paper, JC, SCA and BPNN models are used to characterize the flow behavior of GW103K magnesium alloy during hot deformation. By calculating the values of R, RMSE, and RE between experimental and predicted stress, the predictive ability and fit accuracy of the three models are assessed. The microstructural evolution of GW103K magnesium alloy during hot deformation is also investigated by electron back-scattered diffraction (EBSD).

## 2. Materials and Methods

The experimental material was taken from an as-cast cuboid ingot. GW103K magnesium alloy is a high-strength, heat-resistant, rare earth magnesium alloy. Its chemical composition (wt.%) is listed in Table 1.

**Figure 1:**
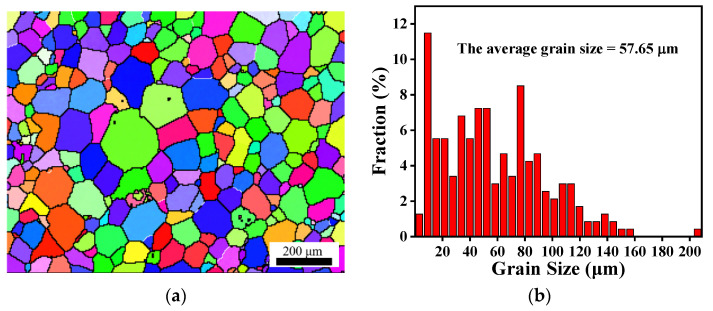
The EBSD micrograph of GW103K alloy before deformation (**a**) inverse pole figure (IPF); (**b**) statistical diagram of grain size.

In order to determine the hot compression behavior of GW103K magnesium alloy, uniaxial compression tests with a strain rate range of 0.001–1 s^−1^ and a temperature range of 623–773 K were conducted using a Gleeble−3500 thermo-mechanical simulator. Cylindrical compression specimens were designed with dimensions of Φ10 mm × 15 mm. To reduce friction between the anvil and the specimen, tantalum foil and graphite were used as a lubricant between the specimen and the anvil during compression. The test sample was heated to deformation temperatures at a rate of 5 K/s and soaked for 180 s for uniform heating. In order to retain the microstructure of the samples, water quenching was performed immediately after compression. For subsequent EBSD studies, the observed plane was sectioned in the center, parallel to the compression direction. After mechanical grinding, the specimen was electropolished in an etchant solution of 70 mL ethanol, 6 mL H_2_O, 4 mL acetic acid, and 4.4 g picric acid at 25 V for 90 s. The EBSD data were analyzed using Channel 5 software and acquired by covering an area of 568 μm × 426 μm with a step of 1.5 μm. Generally speaking, LAGBs and HAGBs are defined by grain boundaries with a misorientation angle of 2°–15° and >15°, respectively [25]. In this paper, the LAGBs are marked by white lines; meanwhile, the HAGBs are marked by black lines in the EBSD images. The grains with HAGBs were used to calculate the grain size. In addition, the grain average misorientation (GAM) map was constructed to characterize “recrystallized”, “substructured” and “deformed” grains in accordance with Channel 5 software [26,27,28]. The initial microstructure of GW103K magnesium alloy before compression is characterized by EBSD (Figure 1). It can be seen that the average grain size is 57.65 μm with a random orientation and an approximately equiaxed grain structure.

## 3. Results and Discussion

### 3.1. Flow Softening Mechanism

**Figure 2:**
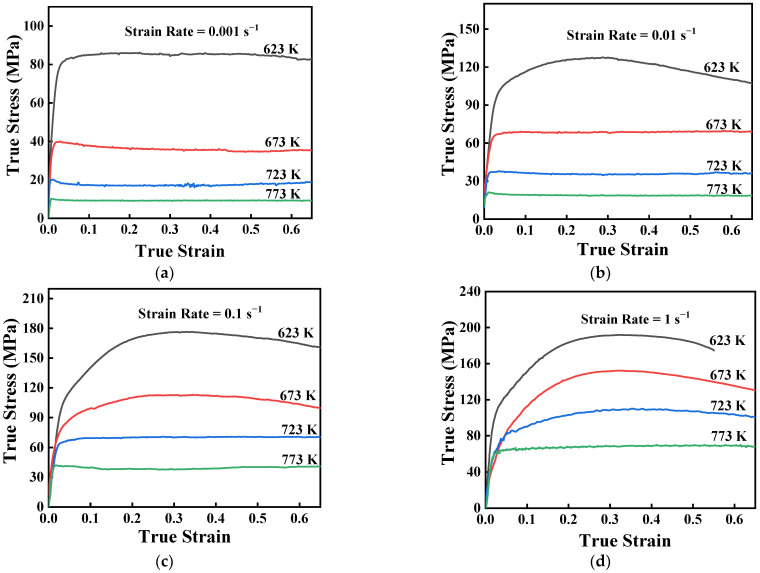
True strain-stress curves of GW103K Mg alloy under different strain rates: (**a**) 0.001 s^−1^; (**b**) 0.01 s^−1^; (**c**) 0.1 s^−1^; and (**d**) 1 s^−1^.

The true stress–strain curves of GW103K Mg alloy at the strain rate and temperature ranges of 0.001–1 s^−1^ and 623–773 K are illustrated in Figure 2. It is apparent that flow stress decreases with increased temperature and decreased strain rate. The thermal activation is strengthened when the temperature is increased. Accordingly, the critical shear stress decreases, which is beneficial to the movement of dislocations. Similarly, an increased strain rate shortens the time of the softening action and leads to the accumulation of dislocations in the deformation process. Moreover, flow stress rises to a peak value, and flow softening appears on further straining in all test conditions. The curves of flow stress can be roughly divided into three stages. Each stage is mainly managed by the competition between the work-hardening and -softening mechanisms [29]. Initially, the flow stress climbs rapidly with the increasing strain, and work hardening is the dominant mechanism. Secondly, as the strain reaches the critical value for DRX or DRV, stress reaches a peak value. Finally, a balance between work hardening and softening is dynamically realized, and a steady-state flow is achieved.

Referring to Figure 2, there are three different rheological curves. For example, the flow stress rapidly reaches the maximum value and remains constant with strain increasing at 673 K/0.01 s^−1^. This is an obvious behavior of DRV, which indicates that DRV is the predominant softening mechanism in this hot compression condition. At 773 K/0.01 s^−1^, the flow curves initially rise to the peak stress, then decrease and eventually reach a steady state. This is attributed to the occurrence of DRX leading to microstructural reconstitution. Furthermore, at 623 K and 0.01 s^−1^, the peak stress behavior is evident without any steady-state flow stress up to the maximum strain. This phenomenon is an indication of incomplete DRX [30].

### 3.2. Johnson-Cook Constitutive Model

Due to its simple form, the JC model has been widely used for various materials. The basic JC equation is expressed as follows:

(1) $$\{\begin{array}{c} \sigma =\left(A+B\varepsilon^{n}\right)\left(1+Cln{\dot{\varepsilon }}^{*}\right)\left(1-T^{*}{}^{m}\right)\\ {\dot{\varepsilon }}^{*}=\dot{\varepsilon }/\dot{\varepsilon_{0}}\\ T^{*}=\left(T-T_{r}\right)/\left(T_{m}-T_{r}\right) \end{array},$$

where σ, ε, $\dot{\varepsilon }$, ${\dot{\varepsilon }}_{0}$, T, $T_{r}$, and $T_{m}$ represent the flow stress, strain, strain rate, reference strain rate, temperature, reference temperature, and melting point, respectively, A represents the yield stress under reference conditions, B, n, C, and m are material constants, ${\dot{\varepsilon }}^{*}$ represents non-dimensional strain rate, and $T^{*}$ represents non-dimensional temperature. The first term of the JC model indicates the strain hardening effect; the second and third terms reflect the strain rate hardening and the thermal softening effect, respectively.

In this research, the melting point of GW103K magnesium alloy is 919 K. The yield stress A is 64.74 MPa under reference conditions (623 K and 0.001 s^−1^). At reference temperature and reference strain rate, the natural logarithm of both sides of Equation (1) is taken. The values of *n* and *B* are obtained from the slope and intercept of ln(σ−A) vs. lnε, respectively.

**Figure 3:**
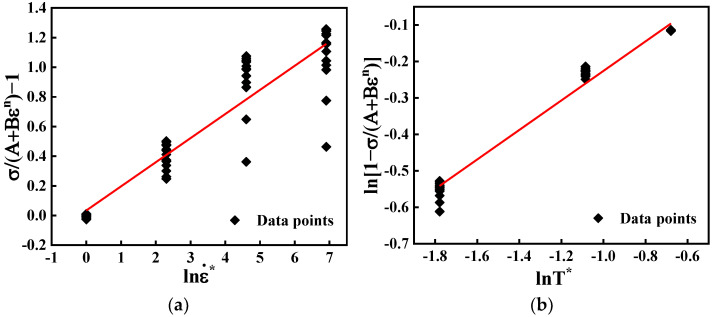
Relationship between: (**a**) $\sigma /\left(A+B\varepsilon^{n}\right)-1$ and $\ln{\dot{\varepsilon }}^{*}$; (**b**) $\ln[1-\sigma /\left(A+B\varepsilon^{n}\right)]$ and $\ln T^{*}$.

When the temperature is 623 K, the material constant *C* can be taken from the gradient of $\left[\sigma /\left(A+B\varepsilon^{n}\right)-1\right]$ against $\ln{\dot{\varepsilon }}^{*}$ (Figure 3a). Similarly, at the reference strain rate of 0.001 s^−1^, the value of *m* can be acquired by calculating the average slope between $\ln\left(1-\sigma /\left(A+B\varepsilon^{n}\right)\right)$ and $\ln T^{*}$ (Figure 3b). The JC constitutive equation for GW103K can be obtained as follows:

(2) $$\{\begin{array}{c} \sigma =\left(64.74+19.917\varepsilon^{-0.0111}\right)\left(1+0.1626\ln{\dot{\varepsilon }}^{*}\right)\left(1-T^{*}{}^{0.40516}\right)\\ {\dot{\varepsilon }}^{*}=\dot{\varepsilon }/0.001\\ T^{*}=\left(T-623\right)/296 \end{array}.$$

### 3.3. Strain-Compensated Arrhenius Model

The Arrhenius equation proposed by Sellers and Tegart is shown in Equation (3):

(3) $$\{\begin{array}{c} \dot{\varepsilon }=AF\left(\sigma \right)exp\left(-Q/RT\right)\;\;\;\;\;\;\;\;\;\;\;\;\;\;\;\;\;\;\;\;\;\;\;\;\;\;\;\;\;\\ F\left(\sigma \right)=\{\begin{array}{c} \sigma^{m}\;\;\;\;\;\;\;\;\;\;\;\;\;\;\;\;\;\;\;\;\;\;\;\;\;\;\left(\alpha \sigma <0.8\right)\\ \exp\left(\beta \sigma \right)\;\;\;\;\;\;\;\;\;\;\;\;\;\;\;\;(\alpha \sigma >1.2)\\ {\left[\sinh\left(\alpha \sigma \right)\right]}^{n}\;\;\;\;\;\;\;\;\;\;\left(For\;all\;\alpha \sigma \right) \end{array}\; \end{array},$$

where σ, *Q*, $\dot{\varepsilon }$, *T*, and R are the true stress (MPa), the activation energy (J/mol), the strain rate (s^−1^), the deformation temperature (K), and the universal gas constant (8.3145 J/(mol·K)), respectively; A, m, α, β, and n are the material constants, α=β/m.

**Figure 4:**
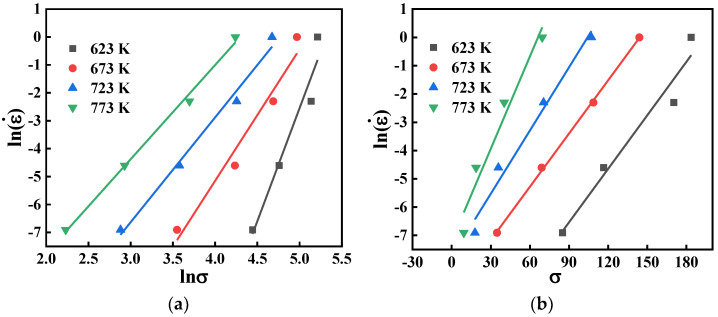
Relationship between: (**a**) ln$\dot{\varepsilon }$ and lnσ; (**b**) ln$\dot{\varepsilon }$ and σ.

Subsequently, the values of *m*, β, n can be retrieved after taking logarithms of Equation (3), i.e., $m=\partial \ln\dot{\varepsilon }/\partial \ln\sigma$, $\beta =\partial \ln\dot{\varepsilon }/\partial \sigma$, and $n=\partial \ln\dot{\varepsilon }/\partial \ln\left[\sinh\left(\alpha \sigma \right)\right]$. Figure 4 shows the relationship between $\ln\dot{\varepsilon }$ vs. lnσ and $\ln\dot{\varepsilon }$ vs. σ. It can be seen that $\ln\dot{\varepsilon }$ is linearly related to lnσ and σ at the same temperature. When ε=0.5, the mean values of m and β are computed as 4.9851 and 0.0773, respectively.

**Figure 5:**
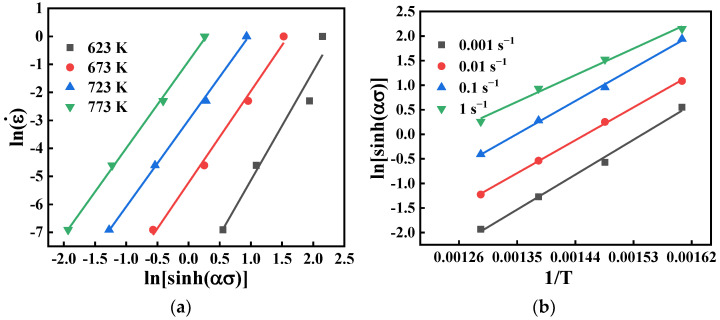
Relationship between: (**a**) $\ln\dot{\varepsilon }$ and ln[sinh(ασ)]; (**b**) ln[sinh(ασ)]  and 1/T.

For all stress levels, the logarithm of both sides of Equation (3) was taken. The value of *n* is the average gradient of the lines in Figure 5a. For the given strain rate conditions, Q can be calculated from Figure 5b. Finally, the average values of n and Q are 3.3436 and 200136.2775 J/mol, respectively.

In order to calculate parameter A, the Zener–Hollomon parameter (Z) is utilized, which is expressed as follows:

(4) $$Z=\dot{\varepsilon }\exp\left(Q/RT\right),$$

**Figure 6:**
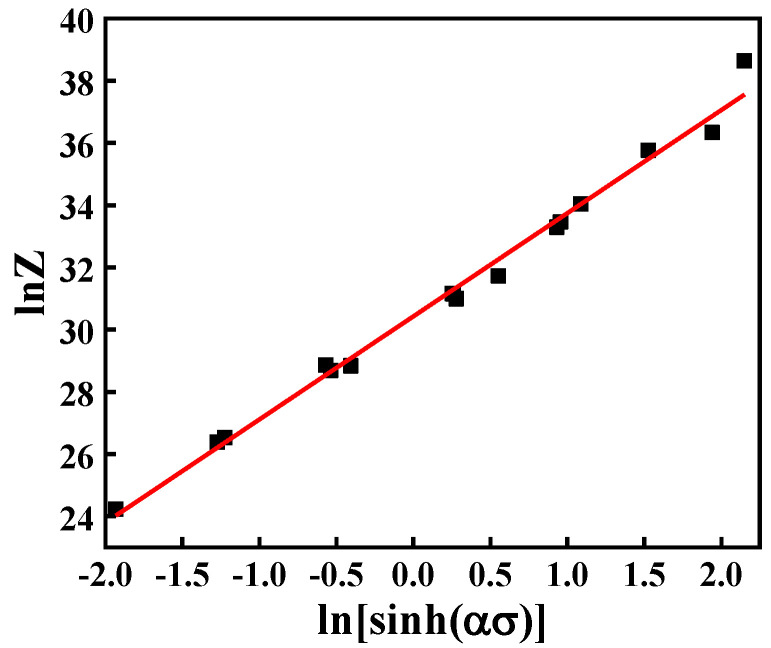
Relationship between lnZ and ln[sinh(ασ)].

Combining Equations (3) and (4), and taking the logarithm of both sides, Equation (5) is derived. It can be seen that lnA is the intercept of the line in Figure 6. The value of lnA is 30.4293.

(5) lnZ=nlnsinh(ασ)+lnA.

**Figure 7:**
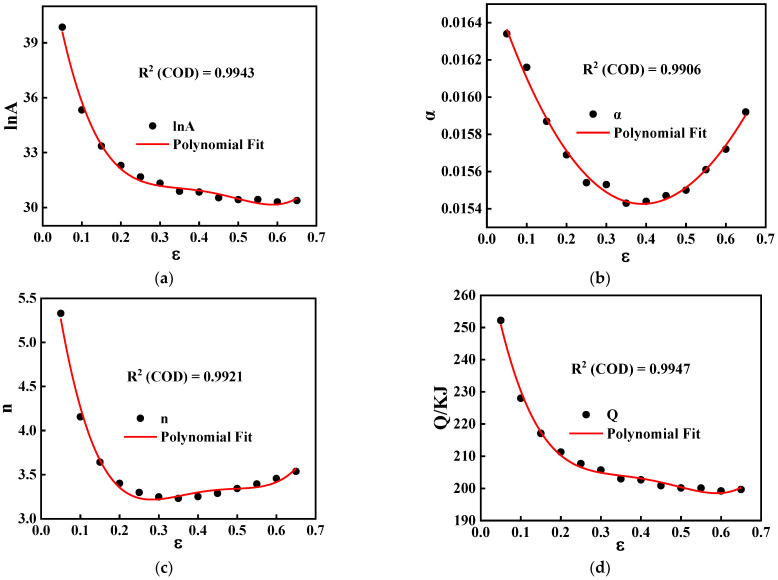
Relationship between: (**a**) lnA and ε; (**b**) α and ε; (**c**) n and ε; (**d**) Q and ε.

In the original Arrhenius equation, stress (σ) only considers the effects of strain rate and temperature, whereas many studies have shown that material constants depend on strain. Therefore, to predict flow stress more accurately, strain should be introduced into the constitutive equation. In this paper, the material parameters under strains from 0.05 to 0.65 were obtained by repeating the above calculation. Figure 7 shows the relationship between lnA, α, n, Q, and strain (ε).

The SCA constitutive model of GW103K was established after polynomial fitting. The final mathematical is expressed as Equation (6):

(6) $$\{\begin{array}{c} \dot{\varepsilon }=A\left(\varepsilon \right)\sinh{\left[\alpha \left(\varepsilon \right)\sigma \right]}^{n\left(\varepsilon \right)}\exp\left[-Q\left(\varepsilon \right)/RT\right]\\ \ln A=45.4664-140.9999\varepsilon +520.6682\varepsilon^{2}-848.9962\varepsilon^{3}+503.4188\varepsilon^{4}\\ \alpha =0.0167-0.0066\varepsilon +0.0094\varepsilon^{2}-0.0016\varepsilon^{3}\\ n=6.8210-37.5314\varepsilon +140.7247\varepsilon^{2}-224.6776\varepsilon^{3}+131.0479\varepsilon^{4}\\ Q=282326.8207-754826.2673\varepsilon +2.7681\times 10^{6}\varepsilon^{2}-4.5122\times 10^{6}\varepsilon^{3}+2.6793\times 10^{6}\varepsilon^{4} \end{array}$$

### 3.4. BP Neural Network Model

**Figure 8:**
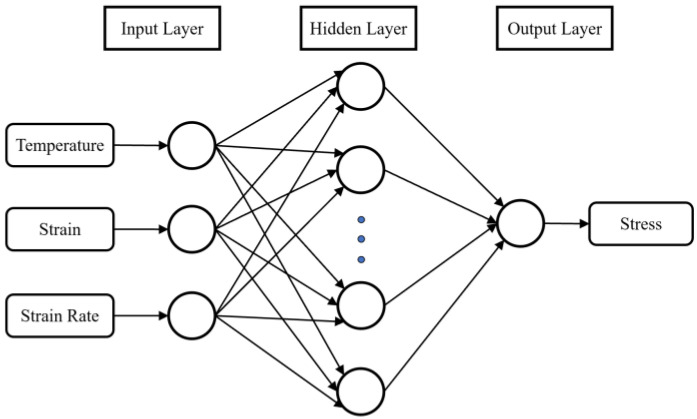
Schematic structure of the feed-forward BPNN.

In this paper, a feed-forward back-propagation neural networks (BPNN) model was constructed to investigate the flow behavior of GW103K alloy (Figure 8). MATLAB platform was applied to train and test the BPNN model. The typical BPNN model contains an input layer, a hidden layer, and an output layer. The deformation temperature, strain, and strain rate were chosen as input neurons. Meanwhile, the output layer neuron signified the flow stress. The number of hidden layer neurons was 15. To acquire greater generalization performance, Bayesian regularization (BR) was chosen to be the training algorithm. The maximum epoch and the minimum gradient were set as 1000 and 1.0 × 10^−7^, respectively.

A total of 444 samples were picked from the true strain–stress curves to train and test the BPNN. Both input and output data should be normalized before training the network. In order to eliminate the influence of features with large variance, it is necessary to normalize the data matrix. Therefore, the original data were normalized using Equation (7), and all the transformed data can also be returned to their original value after the training.

(7) $$x_{i}=\frac{X_{i}-X_{min}}{X_{max}-X_{min}},$$

where $X_{i}$ represents the practical data and $x_{i}$ represents the normalized data, and $X_{max}$ and $X_{min}$ represent the maximum and minimum values, respectively. Additionally, the mean square error (MSE) was used to check the ability of the architecture. The criterion of MSE can be expressed as Equation (8):

(8) $$\mathrm{MSE}=\frac{1}{n}\sum_{i=1}^{n}{\left(y_{i}-y_{i}^{*}\right)}^{2},$$

where, $y_{i}$ and $y_{i}^{*}$ are the calculated value and experiment value of the output neuron i, respectively.

### 3.5. Comparison of Three Models

In order to verify and compare the predictability and stability of the three constructed models, the values of R, RMSE, and RE and were calculated based on Equations (9)–(11), respectively.

(9) $$R=\frac{\sum_{i=1}^{N}\left(E_{i}-\bar{E}\right)\left(P_{i}-\bar{P}\right)}{\sqrt{\sum_{i=1}^{N}{\left(E_{i}-\bar{E}\right)}^{2}\sum_{i=1}^{N}{\left(P_{i}-\bar{P}\right)}^{2}}},$$

(10) $$\mathrm{RMSE}=\sqrt{\frac{1}{N}\sum_{i=1}^{N}{\left(E_{i}-P_{i}\right)}^{2}},$$

(11) $$\mathrm{RE}=\frac{P_{i}-E_{i}}{E_{i}},$$

where $E_{i}$ represents the experimental stress, $P_{i}$ represents the predicted stress, $\bar{E}$ represents the average experimental stress, $\bar{P}$ represents the average predicted stress, and N signifies the number of data points.

**Figure 9:**
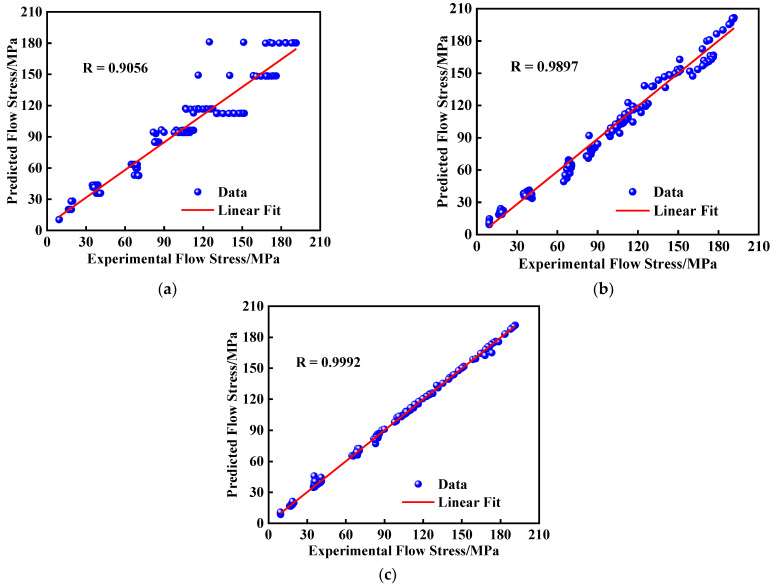
Correlation between the predicted and experimental flow stress by: (**a**) JC model; (**b**) SCA model; (**c**) BPNN model.

Figure 9 shows the correlation between the predicted and experimental values of the three models. The R values for the JC, SCA, and BPNN models were 0.9056, 0.9897, and 0.9992. The closer R is to 1, the better the linear fitting is. Additionally, the RMSE values of JC, SCA, and BPNN were 12.865 MPa, 9.407 MPa, and 1.506 MPa, respectively. These results indicate that the BPNN model has more successful predictability than the JC and the SCA models.

**Figure 10:**
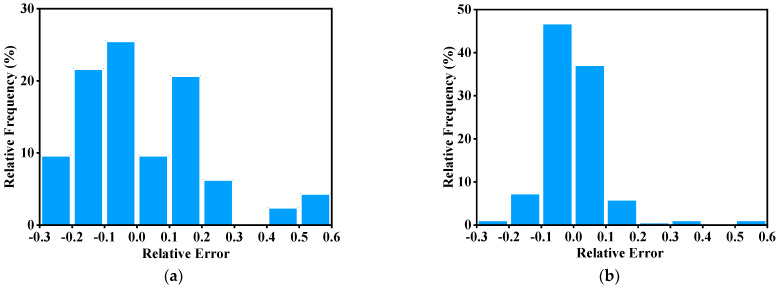

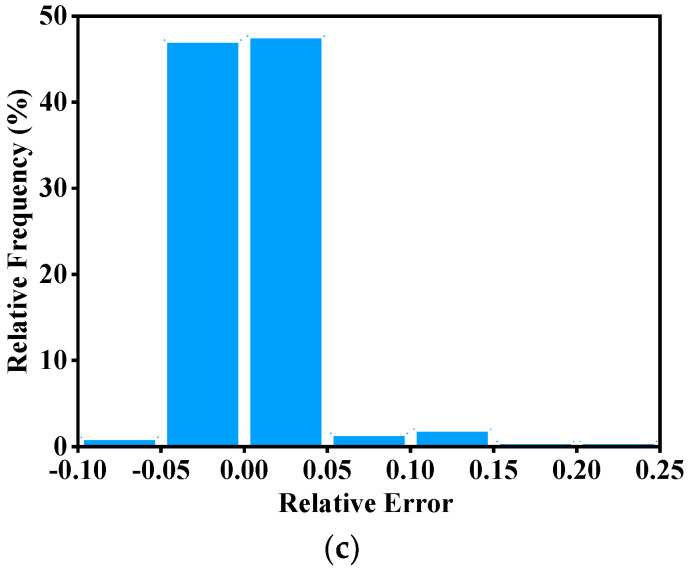
The relative frequency of RE from (**a**) JC model; (**b**) SCA model; (**c**) BPNN model.

In addition, Figure 10 illustrates the relative frequency of the relative errors for the three models. It can be seen that the relative errors of the JC and SCA models are dispersed, while the relative error of the BPNN model within ±0.05 is more than 90%. Meanwhile, the mean values and standard deviation of RE from the three models are listed in Table 2. Although the mean value of RE for the SCA model is the smallest, the standard deviation is more than that of the BPNN model. Therefore, the results prove that the BPNN model has better predictability and accuracy in describing flow behavior than the other two models for GW103K.

### 3.6. Microstructural Evolution

**Figure 11:**
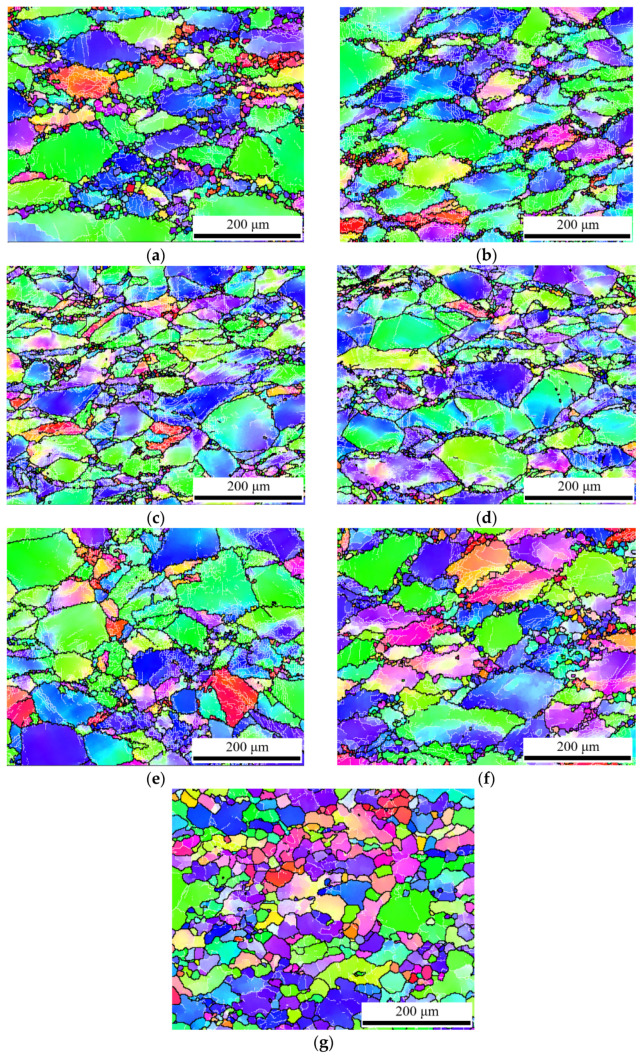
The EBSD micrographs of samples:(**a**) 673 K, 0.001 s^−1^; (**b**) 673 K, 0.01 s^−1^; (**c**) 673 K, 0.1 s^−1^; (**d**) 673 K, 1 s^−1^; (**e**) 623 K, 0.01 s^−1^; (**f**) 723 K, 0.01 s^−1^; (**g**) 773 K, 0.01 s^−1^.

The EBSD micrograms of experimental samples are shown in Figure 11, which are used to explore the softening mechanism (DRX and/or DRV) of GW103K magnesium alloy. It can be observed clearly that, compared with Figure 1a, the original grains are gradually elongated perpendicular to the compression direction, and some fine grains are generated along the old grain boundaries [31]. For magnesium alloys with low SFE, it is difficult for dislocation climb and cross slip to occur. As a result, the low dynamic recovery rate and the high dislocation density of substructure can promote the formation of dynamic recrystallization. The occurrence of DRX in Mg alloy has been previously validated in many studies [32,33,34].

Figure 11a–d show the microstructures at a temperature of 673 K and strain rates of 0.001 s^−1^, 0.01 s^−1^, 0.1 s^−1^, and 1 s^−^^1^, respectively. A typical necklace-type microstructure is visible from the grain boundaries under these conditions [35]. The size and proportion of new strain-free grains increased with a decrease in strain rate. This is because the lower the strain rate is, the more deformation time the material has. Then, the DRX grain content is higher, and new grain sizes become larger. In addition, Figure 11b,e,f,g show the microstructures at a strain rate of 0.01 s^−1^ and temperatures of 623 K, 673 K, 723 K, and 773 K, respectively. It is noted that the amount of DRX grains and grain size increased gradually with increasing temperature. In particular, the recrystallized grains grew, homogenized, and completely replaced the original grains when the temperature rose to 773 K (in Figure 11g). This is due to the fact that DRX is a thermal activation process. The critical shear stress (CSS) decreases and the slip system increases with increasing temperature. Moreover, the dislocation movement and grain boundary migration are accelerated at an elevated temperature.

**Figure 12:**
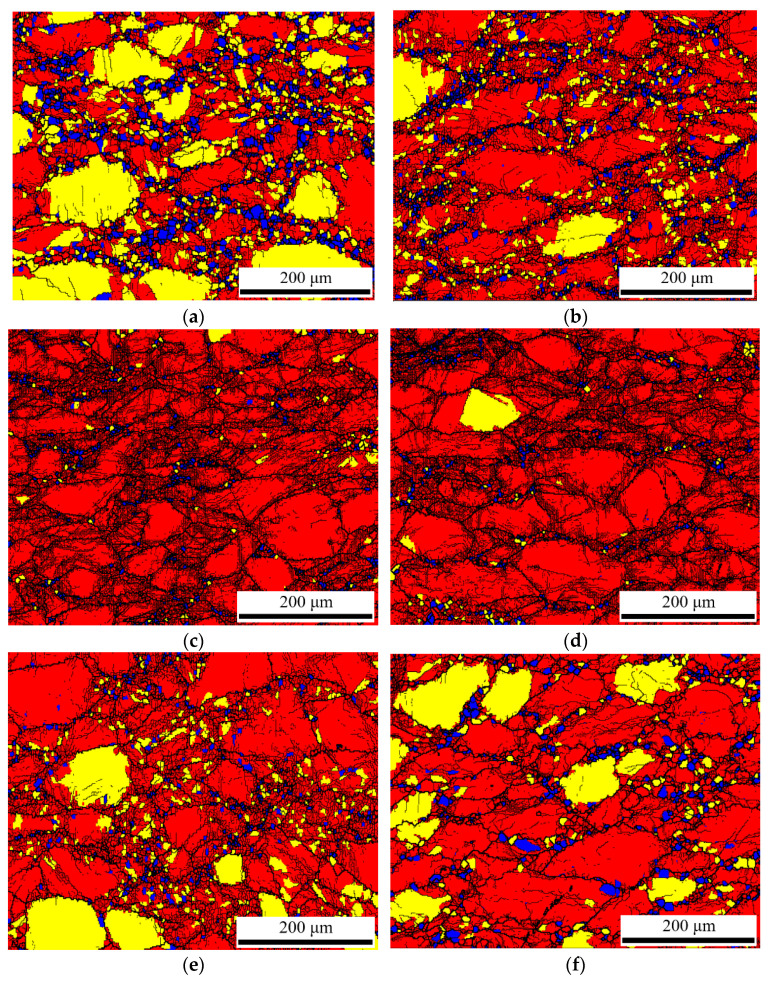

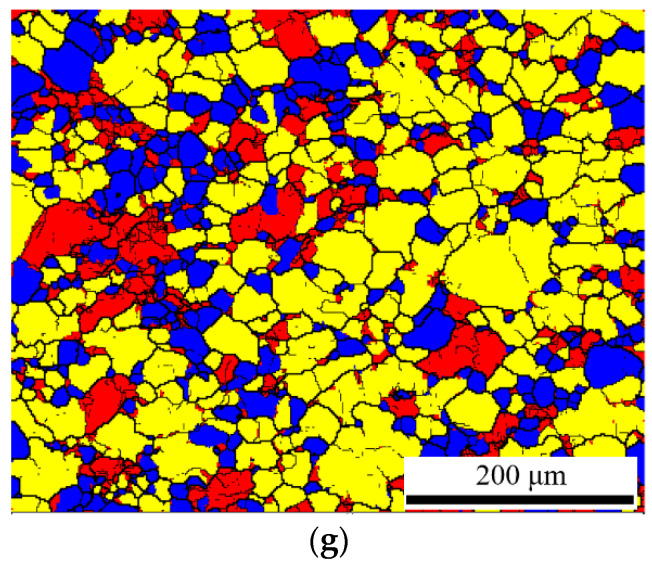
GAM maps of recrystallized, substructured, and deformed grains at conditions: (**a**) 673 K, 0.001 s^−1^; (**b**) 673 K, 0.01 s^−1^; (**c**) 673 K, 0.1 s^−1^; (**d**) 673 K, 1 s^−1^; (**e**) 623 K, 0.01 s^−1^; (**f**) 723 K, 0.01 s^−1^; (**g**)773 K, 0.01 s^−1^.

In order to understand the distribution of dynamically recrystallized grains more clearly, grain average misorientation (GAM) maps were constructed (shown in Figure 11), which distinguish recrystallized (blue), substructured (yellow) and deformed (red) grains via Channel 5 software. Figure 12a–f show that DRX grains (blue color) are distributed along grain boundaries, which corresponds to Figure 11a–f. As for Figure 12g, the reason why the content of DRX is not 100% is that dynamic recrystallization behavior is not a transient process. With the increase in strain, the DRX grains formed in the early stage are extruded and deformed, and new DRX occurs again. According to EBSD maps and stress–strain curves, the microstructure can reflect the rheological process. For instance, there are some DRX grains along grain boundaries, but the original grains and their internal substructures occupied the majority under the condition of 673 K and 0.01 s^−1^, which demonstrates that the dominant softening mechanism is DRV at this deformation condition. In addition, the DRX grains homogenized and completely replaced the original grains at 773 K and 0.01 s^−1^, which reveals that DRX is the main softening mechanism. These results are consistent with the above stress–strain analysis.

**Figure 13:**
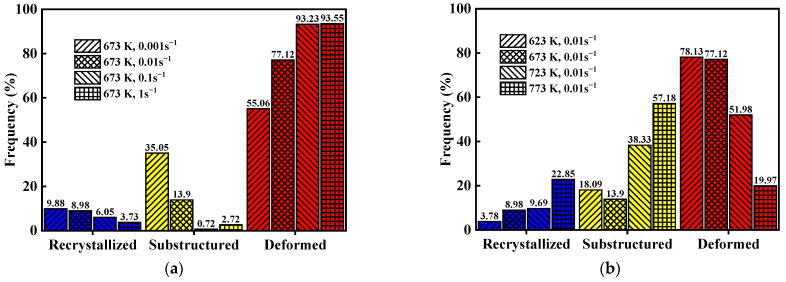
Comparison of recrystallized, substructured, and deformed grains under different hot working conditions (**a**) 0.001 s^−1^, 0.01 s^−1^, 0.1 s^−1^, 1 s^−1^ under 673 K; (**b**) 623 K, 673 K, 723 K, 773 K under 0.01 s^−1^.

According to Figure 12, the frequency of recrystallized, substructured and deformed grains under different conditions are obtained (shown in Figure 13). Figure 13a shows that the area fraction of recrystallized grains and substructured grains reduces, while the proportion of deformed grains increases with an increase in strain rate at the same temperature. Meanwhile, Figure 13b illustrates that the area fraction of recrystallized grains and substructured grains increases, while the proportion of deformed grains decreases with an increase in temperature. Consequently, it can be concluded that the formation of dynamic recrystallization is more easily promoted by a high temperature and a low strain rate for GW103K magnesium alloy under hot compression.

## 4. Conclusions

The high-temperature rheological behavior of GW103K magnesium alloy at a strain rate of 0.001–1 s^−1^ and temperature ranges of 623–773 K was investigated using thermal compression tests. JC, SCA, and BPNN models were employed to investigate its flow behavior. Moreover, the microstructure evolution of GW103K alloy was also researched. The specific conclusions are as follows.

1. The flow stress of GW103K is significantly correlated with strain rate and temperature; flow stress value increases when strain rate is raised and temperature is reduced.
2. Comparatively, the JC and SCA models are inadequate to describe the flow behavior of GW103K alloy. The BPNN model has higher accuracy and efficiency than the other two models in predicting the flow behavior of GW103K magnesium alloy.
3. It has been proved that DRX is more likely to take place with increasing temperature and decreasing strain rate. Meanwhile, the size of recrystallized grains will also be larger under higher temperature and/or lower strain rate.

## Figures and Tables

**Table 1:** Chemical composition (wt.%).

Gd	Y	Zr	Mg
10.3	3.3	0.4	Bal

**Table 2:** The mean value and standard deviation of RE for the three models.

Model	Mean Value	Standard Deviation
JC	0.0154	0.1878
SCA	0.0006	0.0992
BPNN	0.0053	0.0354

## Data Availability

Not applicable.

## References

[1] Kulekci M.K. (2008). Magnesium and its alloys applications in automotive industry. Int. J. Adv. Manuf. Technol..

[2] Joost W.J., Krajewski P.E. (2017). Towards magnesium alloys for high-volume automotive applications. Scr. Mater..

[3] Li L., Zhang X.M. (2011). Hot compression deformation behavior and processing parameters of a cast Mg-Gd-Y-Zr alloy. Mater. Sci. Eng. A.

[4] Hort N., Huang Y.D., Kainer K.U. (2006). Intermetallics in magneisum alloys. Adv. Eng. Mater..

[5] Hajari A., Morakabati M., Abbasi S.M., Badri H. (2016). Constitutive modeling for high-temperature flow behavior of Ti-6242S alloy. Mater. Sci. Eng. A.

[6] Zheng T.T., Lin D.J., Zeng X.Q., Ding W.J. (2016). Hot compressive deformation behaviors of Mg–10Gd–3Y–0.5Zr alloy. Prog. Nat. Sci. Mater..

[7] Lin Y.C., Chen X. (2011). A critical review of experimental results and constitutive descriptions for metals and alloys in hot working. Mater. Design..

[8] Johnson G.R., Cook W.H. (1983). A constitutive model and data for metals subjected to large strains, high strain rates and high temperatures. Eng. Fract. Mech..

[9] Sellars C.M., Mctegart W.J. (1966). On the mechanism of hot deformation. Acta Metall. Mater..

[10] Roy M., Maijer D., Dancoine L. (2012). Constitutive behavior of as-cast A356. Mater. Sci. Eng. A.

[11] Bisht A., Yadav V., Suwas S., Dixit U.S. (2018). Deformation Behavior of AM30 Magnesium Alloy. J. Mater. Eng. Perform..

[12] Zhou T.F., Wu J.J., Liang Z.Q., Che J.T., Zhang Y.C., Wang X.B. (2017). A novel constitutive model for Ti-6Al-4V alloy based on dislocation pile-up theory. Mater. Sci. Technol..

[13] Haghdadi N., Zarei-Hanzaki A., Abedi R.H. (2012). The flow behavior modeling of cast A356 aluminum alloy at elevated temperatures considering the effect of strain. Mater. Sci. Eng. A.

[14] Ashtiani H.R.R., Shahsavari P. (2016). Strain-dependent constitutive equations to predict high temperature flow behavior of AA2030 aluminum alloy. Mech. Mater..

[15] Slooff F.A., Zhou J., Duszczyk J., Katgerman L. (2007). Constitutive analysis of wrought magnesium alloy Mg–Al4–Zn1. Scr. Mater..

[16] Arjomandi M., Sadati S.H., Khorsand H., Abdoos H. (2008). Austenite formation temperature prediction in steels using an artificial neural network. Defect Diff. Forum.

[17] Abbasi-Bani A., Zarei-Hanzaki A., Pishbin M.H., Haghdadi N. (2014). A comparative study on the capability of Johnson–Cook and Arrhenius-type constitutive equations to describe the flow behavior of Mg–6Al–1Zn alloy. Mech. Mater..

[18] Adarsh S.H., Sampath V. (2020). Prediction of high temperature deformation characteristics of an Fe-based shape memory alloy using constitutive and artificial neural network modelling. Mater. Today Commun..

[19] Wang M.H., Wang G.T., Wang R. (2016). Flow stress behavior and constitutive modeling of 20MnNiMo low carbon alloy. J. Cent. South Univ..

[20] Sandlobes S., Friak M., Zaefferer S., Dick A., Yi S., Letzig D., Pei Z., Zhu L.F., Neugebauer J., Raabe D. (2012). The relation between ductility and stacking fault energies in Mg and Mg-Y alloys. Acta Mater..

[21] Marchattiwar A., Sarkar A., Chakravartty J.K., Kashyap B.P. (2013). Dynamic Recrystallization during Hot Deformation of 304 Austenitic Stainless Steel. J. Mater. Eng. Perf..

[22] Gourdet S., Montheillet F. (2000). An experimental study of the recrystallization mechanism during hot deformation of aluminium. Mater. Sci. Eng. A.

[23] Ion S.E., Humphreys F.J., White S.H. (1982). Dynamic recrystallisation and the development of microstructure during the high temperature deformation of magnesium. Acta Metal..

[24] Maghsoudi M.H., Zarei-Hanzaki A., Changizian P., Marandi A. (2014). Metadynamic recrystallization behavior of AZ61 magnesium alloy. Mater. Des..

[25] Sumit G., Madan P., Mahesh C.S., Pasi P. (2021). Characteristics of dynamic softening during high temperature deformation of CoCrFeMnNi highentropy alloy and its correlation with the evolving microstructure and micro-texture. J. Mater. Res. Technol..

[26] Chen B.R., Yeh A.C., Yeh J.W. (2016). Effect of one-step recrystallization on the grain boundary evolution of CoCrFeMnNi high entropy alloy and its subsystems. Sci. Rep..

[27] Thirathipviwat P., Song G., Jayaraj J., Bednarcik J., Wendrock H., Gemming T., Freudenberger J., Nielsch K., Han J. (2019). A comparison study of dislocation density, recrystallization and grain growth among nickel, FeNiCo ternary alloy and FeNiCoCrMn high entropy alloy. J. Alloys Comp..

[28] Masoumi M., Herculano L.F.G., Almeida A.A., Beres M., De Abreu H.F.G. (2016). Texture study across thickness of API X70 steel after hot deformation and different posttreatments. JOM.

[29] Solhjoo S. (2010). Determination of critical strain for initiation of dynamic recrystallization. Mater. Des..

[30] Guo N.N., Wang L., Luo L.S., Li X.Z., Chen R.R., Su Y.Q., Guo J.J., Fu H.Z. (2016). Hot deformation characteristics and dynamic recrystallization of the MoNbHfZrTi refractory high-entropy alloy. Mater. Sci. Eng. A.

[31] Sakai T., Jonas J.J. (1984). Dynamic recrystallization: Mechanical and microstructural considerations. Acta Metal..

[32] Galiyev A., Kaibyshev R., Gottstein G. (2001). Correlation of plastic deformation and dynamic recrystallization in magnesium alloy Zk60. Acta Mater..

[33] Takigawa Y., Honda M., Uesugi T., Higashi K. (2008). Effect of Initial Grain Size on Dynamically Recrystallized Grain Size in AZ31 Magnesium Alloy. Mater. Trans..

[34] Hadadzadeh A., Mokdad F., Wells M.A., Chen D.L. (2018). Modeling dynamic recrystallization during hot deformation of a cast-homogenized Mg-Zn-Zr alloy. Mater. Sci. Eng. A.

[35] Martin E., Jonas J.J. (2010). Evolution of microstructure and microtexture during the hot deformation of Mg–3% Al. Acta Mater..

